# Impacts of clinicopathologic and operative factors on short-term and long-term survival in renal cell carcinoma with venous tumor thrombus extension: a multi-institutional retrospective study in Japan

**DOI:** 10.1186/1471-2407-13-447

**Published:** 2013-10-02

**Authors:** Masanori Hirono, Mikio Kobayashi, Tomoyasu Tsushima, Wataru Obara, Nobuo Shinohara, Keiichi Ito, Masatoshi Eto, Tatsuya Takayama, Yasuhisa Fujii, Masaharu Nishikido, Go Kimura, Takeshi Kishida, Masayuki Takahashi, Noriomi Miyao, Yukio Naya, Takashige Abe, Tomoaki Fujioka, Kazuto Ito, Seiji Naito

**Affiliations:** 1 Department of Urology, Gunma University Graduate School of Medicine, Maebashi, Japan; 2 Division of Urology, Isesaki Municipal Hospital, 12-1, Tsunatori-hon-machi, 372-0817 Isesaki, Gunma, Japan; 3 Division of Urology, Okayama Medical Center, Okayama, Japan; 4 Department of Urology, Iwate Medical University School of Medicine, Morioka, Japan; 5 Department of Renal and Genitourinary surgery, Graduate School of Medicine, Hokkaido University, Sapporo, Japan; 6 Department of Urology, National Defense Medical College, Tokorozawa, Japan; 7 Department of Urology, Faculty of Life Sciences Kumamoto University, Kumamoto, Japan; 8 Department of Urology, Kyushu University Faculty of Medicine, Fukuoka, Japan; 9 Department of Urology, Hamamatsu University School of Medicine, Hamamatsu, Japan; 10 Division of Urology, Cancer Institute Hospital of Japanese Foundation for Cancer Research, Tokyo, Japan; 11 Department of Urology, Tokyo Medical and Dental University, Graduate School of Medical and Dental Sciences, Tokyo, Japan; 12 Department of Urology, Nagasaki University Graduate School of Biomedical Sciences, Nagasaki, Japan; 13 Department of Urology, Nippon Medical School, Tokyo, Japan; 14 Department of Urology, Yokohama City University Hospital, Yokohama, Japan; 15 Department of Urology, Kanagawa Prefectural Cancer Center, Yokohama, Japan; 16 Department of Urology, Tokushima University School of Medicine, Tokushima, Japan; 17 Division of Urology, Muroran City General Hospital, Muroran, Japan; 18 Department of Urology, Chiba University Graduate School of Medicine, Chiba, Japan; 19 Department of Urology, Teikyo University Chiba Medical Center, Ichihara, Japan

**Keywords:** Renal cell carcinoma, Tumor thrombus, Prognostic factors, Overall survival, Cause-specific survival

## Abstract

**Background:**

Although the percentage of patients with renal cell carcinoma (RCC) extending into venous systems is unexpectedly high, the prognostic impact and independency of venous tumor thrombus-related factors on overall survival (OS) remain controversial. Furthermore, the prognostic impact of various clinicopathologic factors including tumor thrombus-related factors on OS may change with elapsed years after the intervention and also with follow-up duration of participants. The aim of the study is to explore independent and universal predictive preoperative and intraoperative clinicopathologic factors on OS in patients with RCC extending into venous systems using subgroup analysis in terms of restricted follow-up duration and yearly-based survivors.

**Methods:**

Between 1980 and 2009, 292 patients diagnosed with RCC with venous tumor thrombus were retrospectively registered for this study. The prognostic impacts of various clinicopathologic and surgical treatment factors including levels of venous thrombus, venous wall invasion status and likelihood of aggressive cytoreductive operation, were investigated using Kaplan-Meier method and following multivariate Cox proportional hazards model for all patients and those still alive at 1, 2, and 3 years of follow-up. To investigate the impact of follow-up duration on the statistical analyses, multivariate logistic regression analyses were used to explore prognostic factors using restricted data until 1, 2, and 3 years of follow-up.

**Results:**

The median follow-up duration was 40.4 months. The 5-year OS was 47.6%. Several independent predictive factors were identified in each subgroup analysis in terms of yearly-based survival and restricted follow-up duration. The presence of tumor thrombus invading to venous wall was independently related to OS in the full-range follow-up data and in survivors at 2 and 3 years of follow-up. Using restricted follow-up data until 1, 2, and 3 years of follow-up, many independent predictive factors changed with follow-up duration, but surgical category could be universal and independent predictive factors.

**Conclusion:**

The most universal factors affecting improvement both in short-term and long-term survivals could be cytoreductive surgery and absence of venous wall invasion. It may mean that feasible aggressive cytoreductive operation following more reliable preoperative imaging for predicting venous wall invasion status would improve OS for patients with RCC extending into venous systems.

## Background

Although the incidence of small and incidentally detected renal cell carcinoma (RCC) has increased, the percentage of patients with tumor thrombus extending into the renal vein (RV) or inferior vena cava (IVC) is unexpectedly high at 4 to 10% of total patients diagnosed with RCC [1-4]. These patients usually need very careful management. Therefore, a very experienced team including urologic surgeons, general surgeons, and sometimes cardiologic surgeons may be essential for perioperative management because there may be a risk of operation-related death at an unacceptable frequency.

Although many clinicians have investigated the impact of tumor thrombus on survival of patients with RCC, controversies surrounding this issue remain [5-9]. In general, predicting prognosis of patients with very advanced stages of cancer is difficult because multifactorial issues are often involved. In the view point of clinicians, it is known that some clinicopathologic factors affect short-term survival while others are related to long-term survival. Controversy regarding the prognostic impact of tumor thrombus in patients with RCC may be at least partly due to the difference in the follow-up duration of the recruited data in the previous studies.

To address the impact of classical clinicopathologic factors, levels of tumor thrombus, venous wall invasion and also likelihood of aggressive cytoreductive operation in patients with RCC with venous thrombus on short-term and long-term overall survival, the present comprehensive univariate and following multivariate statistical analyses were conducted using a multi-institutional data provided by 17 hospitals in which all operations were performed by experienced urologists who are members of the Japanese Society of Renal Cancer.

## Methods

Between October 1980 and March 2009, consecutive 292 patients diagnosed with RCC that extends into the RV, IVC, or right atrium at 17 hospitals belonging to the Japanese Society of Renal Cancer were retrospectively registered in the present study. The year of registration was 1980s, 1990s and 2000s in 8 (2.7%), 136 (46.6%) and 148 (50.7%) patients, respectively. All participants had pathologically confirmed RCC from surgical specimens in patients who underwent operations or from transluminal core-biopsy of the renal tumor, biopsy of metastatic lesions, or aspiration cytology in those who did not undergo radical nephrectomy. All patients underwent a bone scan and chest, abdominal, and pelvic computed tomography (CT) for clinical staging. Ninety one patients with distant metastases were also enrolled in the present study in order to investigate whether cytoreductive surgery was feasible in such patients. The date of last follow-up was August 6, 2009. No patients were treated with molecular-targeted therapy. All pretreatment clinicopathologic data were collected from medical records by urologists in each institution according to the checking sheet for the present research. There was no restricted treatment strategy for the use of interferon or interleukin in adjuvant or salvage settings. There were no restricted follow-up criteria, but blood examinations were done at least once in every 6 months until 5 years of follow-up and in every 6 month thereafter. CT was conducted at least once in every 6 months until 5 years of follow-up and at least annually thereafter, regardless of clinical symptoms. Individual causes of death were judged and recorded by experienced clinical urologists in each institution working in inpatient clinics, most of whom were not associated with the present study.

The levels of tumor thrombus extension were stratified into five categories: (1)intrarenal vein, (2)infrahepatic IVC, (3)suprahepatic IVC, (4)intrapericardial IVC, and (5)intracardiac extension (right atrium) according to the classification proposed by Cummings. Pretreatment prognostic factors included age, clinical symptoms at diagnosis, operative experience in each hospital, performance status (PS) as defined by the Eastern Cooperative Oncology Group, hemoglobin (Hb) level, erythrocyte sedimentation rate (ESR), serum lactate dehydrogenase (LDH) level, calcium (Ca) concentration, C-reactive protein (CRP), immunosuppressive acidic protein (IAP), α2 globulin, and clinical tumor features including lymph node metastasis, distant metastasis and level of tumor thrombus. Pathological prognostic factors included tumor nuclear grade, histopathological subtypes, tumor diameter at origin, perinephric fat invasion, invasion of RV/IVC walls. Invasive status of RV/IVC walls was also judged clinically during operation in some patients undergoing radical nephrectomy, but having been unable to resect thrombus completely. Tumor status and operative management at the tumor origin, tumor thrombus, and metastatic sites were classified into five surgical categories: 1) radical nephrectomy and complete resection of thrombus without metastasis, 2) radical nephrectomy and complete resection of thrombus with metastases that has undergone a cytoreductive surgery, 3) radical nephrectomy and complete resection of thrombus with unresected metastases, 4) radical nephrectomy and incomplete resection of thrombus regardless of metastatic status, and 5) no operation.

Multivariate Cox proportional hazards model was used to explore predictors on overall survival in all 292 participants. To clarify whether prognostic factors change with elapsed postoperative follow-up years, impacts of the above-indicated clinicopathologic factors were investigated for patients who were alive at 1, 2, and 3 years of follow-up.

Furthermore, the prognostic impact of the above general and tumor-related factors were also assessed using restricted data until 1, 2, and 3 years of follow-up in order to investigate the impact of follow-up duration on statistical analyses of prognostic factors. This unique analysis using restricted follow-up data may clarify prognostic factors that affect short-term and/or long-term survival.

All statistical analyses were performed using Dr. SPSS II (SPSS, Inc., Chicago, IL, USA) or Stat Flex (Ver.5.0; Artech Co., Ltd., Osaka, Japan). Cause-specific survival (CSS) and overall survival (OS) were estimated by Kaplan–Meier analysis, and the significance of differences was evaluated by the log-rank test. The above-mentioned candidate prognostic factors were investigated in terms of their relationships with cause-specific death and all-cause death. The cut-offs of continuous clinicopathological factors for Kaplan–Meier analyses were explored by separating patients into binary, tertiary, or quartiles to establish more significant and meticulous separation. If two adjacent subgroups were considered to have an equal predictive value, they were combined. Categorized clinicopathologic factors were also explored in terms of their best cut-lines to establish more significant and meticulous separation. Significant cut-lines for those factors were then explored, and candidates for multivariate analyses were selected and eliminated after considering Spearman’s rank correlation coefficient. The Cox proportional hazard model or multiple logistic regression analysis was used to determine independent and significant predictive factors. To determine independent surrogate factors predictive of OS, a stepwise multiple regression analysis was performed using forward selection. In this analysis, all clinicopathological factors were handled as categorical variables. Differences were considered statistically significant at a *p* value of <0.05.

The ethics review committee of the institution of the chief investigator (Isesaki Municipal Hospital) and the individual institutional review boards of all participating facilities approved this study.

## Results

Of 292 patients with a tumor thrombus that extended into the RV or IVC, 152 (52.1%) had a tumor thrombus within the RV, 101 (34.6%) had a thrombus that extended to the IVC below the hepatic vein (infrahepatic IVC), 20 (6.8%) had a thrombus that extended to the suprahepatic IVC, and 11 (3.8%) had a thrombus that extended to the intracardial IVC or right atrium. Table 1 shows the clinicopathologic features of RCC extending into the venous system as stratified by the level of tumor thrombus. The gender, age, PS, CRP, tumor location, presence or absence of perinephric fat invasion/lymph node metastases/distant metastases, nuclear grade, and pathological tumor subtype were not significantly different among the levels of tumor thrombus. Alternatively, patients with a tumor thrombus within the RV had a lower ESR compared with those with a tumor thrombus extending to the suprahepatic IVC. Patients with a tumor thrombus within the RV or infrahepatic IVC had a lower IAP compared with those that extended to the suprahepatic IVC.

**Table 1:** Clinicopathologic features of renal cell carcinoma extending into the venous system stratified by level of tumor thrombus

	**Level of tumor thrombus**						**Statistical significance (chi-square test or Mann–Whitney U test)**
**Variables**	**Intra-renal vein**	**Infrahepatic IVC**	**Suprahepatic IVC**	**Intrapericardial IVC/ intracardiac extension**	**Unknown**	**Total**	
**n**	148	101	20	11	8	292	
**Gender (n)**							
Male	108	80	15	7	7	217	n.s.
Female	44	21	5	4	1	75	
**Age (years)**							
Mean ± S.D.	63.7 ± 10.8	60.8 ± 10.4	62.3 ± 11.8	60.6 ± 12.5	61.8 ± 11.4	62.4 ± 10.8	n.s.
**Performance status (n)**							
0	105	73	11	5	3	197	n.s.
1	18	11	6	2	2	39	
2	10	2	1	1	0	14	
3	2	4	0	1	0	7	
Unknown	17	11	2	2	3	35	
**ESR (mm)**							
Mean ± S.D.	56.5 ± 44.1	65.4 ± 43.7	81.7 ± 38.6	43.8 ± 57.2	93.3 ± 70.6	62.0 ± 44.6	p < 0.05; intra-renal vein vs. suprahepatic IVC
**CRP (ng/ml)**							
Mean ± S.D.	3.7 ± 4.9	4.0 ± 5.7	5.3 ± 5.6	1.7 ± 1.4	7.2 ± 10.0	3.9 ± 5.4	n.s.
**IAP (ug/ml)**							
Mean ± S.D.	796.9 ± 420.5	794.4 ± 328.2	1020.4 ± 438.7	828.2 ± 372.9	789 ± 475	813.2 ± 391.4	p < 0.05; intra-renal vein, infrahepatic IVC vs. suprahepatic IVC
**Tumor size classification (n)**							
<4 cm	17	5	1	2	0	25	n.s.
4-7 cm	40	22	5	4	2	73	
>7 cm	90	70	11	4	4	179	
Unknown	5	4	3	1	2	15	
**Tumor location (n)**							
Right	72	67	12	9	5	165	n.s.
Left	76	33	8	2	1	120	
Bilateral	2	1	0	0	0	3	
Missing data	2	0	0	0	2	4	
**Perinephric fat invasion (n)**							
No	56	38	12	5	3	114	n.s.
Yes	21	17	3	1	1	43	
Unknown	75	46	5	5	4	135	
**Regional lymph node involvement (n)**							
No	74	29	10	4	1	118	n.s.
Yes	27	23	3	2	6	61	
Unknown	51	49	7	5	1	113	
**Distant metastases (n)**							
No	100	60	12	8	3	183	n.s.
Yes	47	32	4	3	5	91	
Unknown	5	9	4	0	0	18	
**Tumor nuclear grade (n)**							
G1	15	9	2	1	0	27	n.s.
G2	103	52	14	6	4	179	
G3	29	38	4	3	1	75	
Unknown	5	2	0	1	3	11	
**Histopathologic category (n)**							
Clear cell	112	67	17	7	3	206	n.s.
Papillary, chromophobe, others	28	29	3	4	3	67	
Spindle, sarcomatoid	12	5	0	0	2	19	

A total of 196 (67.1%) patients underwent radical nephrectomy and complete resection of thrombus without apparent metastasis, 11 (3.8%) underwent radical nephrectomy, complete resection of thrombus and cytoreductive surgery at metastatic sites, 66 (22.6%) underwent radical nephrectomy and complete resection of thrombus operation and with unresected metastasis, 8 (2.7%) underwent radical nephrectomy and incomplete resection of thrombus, and remaining 11 (3.8%) were unable to undergo operation. Table 2 shows correlations of operative status and metastatic management with clinicopathologic features of participants. Age , tumor size and tumor nuclear grade did not affect operative and metastatic status/management of patients, except for patients classified into surgical category 4 who were younger than those classified into surgical category 1, 3 or 5. Patients who were unable to undergo operation (surgical category 5) had lower PS than those underwent radical nephrectomy and complete resection of thrombus (surgical category 1, 2 or 3). The presence of perinephric fat invasion was significantly lower in patients without metastasis undergoing radical nephrectomy and complete resection of thrombus (surgical category 1) than those undergoing radical nephrectomy and complete resection of thrombus with resected or unresected metastases (surgical category 2 or 3). Patients with non-clear cell subtypes tended to unable to undergo operation than those with clear cell subtype.

**Table 2:** Correlations of operative and metastatic status/management with clinicopathologic features of renal cell carcinoma extending into venous system

**Variables**	**Operative status of RCC extending to the venous system and metastatic status/management**					**Statistical significance (chi-square test or Mann–Whitney U test)**
	**Radical nephrectomy and complete resection of thrombus**			**Radical nephrectomy and incomplete resection of thrombus regardless of metastatic status**	**Abandoned operation**	
	**Without metastasis**	**Existing metastasis and undergoing cytoreductive operation**	**With unresected metastases**			
	**Surgical category 1**	**Surgical category 2**	**Surgical category 3**	**Surgical category 4**	**Surgical category 5**	
**n**	196	11	66	8	11	
**Age (years)**						
Mean ± SD	63.0 ± 11.0	57.5 ± 9.1	61.7 ± 9.8	54.3 ± 5.8	66.8 ± 13.8	p < 0.05; surgical category 4 vs. surgical category 1, 3, 5
**Performance status (n)**						
0	143	7	43	4	0	p < 0.05: surgical category 1, 2, 3 vs. surgical category 5 surgical category 1 vs. surgical category 4
1	22	2	10	1	4	
2	6	1	5	0	2	
3	2	0	3	1	1	
Unknown	23	1	5	2	4	
**Tumor size classification (n)**						
<4 cm	20	0	5	0	0	n.s.
4-7 cm	48	1	19	3	2	
>7 cm	119	10	40	5	5	
Unknown	9	0	2	0	4	
**Perinephric fat invasion (n)**						
No	86	3	20	5	0	p < 0.05: surgical category 1 vs. surgical category 2, 3
Yes	22	4	16	1	0	
Unknown	88	4	30	2	11	
**Tumor nuclear grade (n)**						
G1	18	1	5	3	0	n.s.
G2	129	7	42	1	0	
G3	48	3	19	4	0	
Unknown	1	0	0	0	11	
**Histopathologic category (n)**						
Clear cell	144	10	48	4	0	p < 0.05; : surgical category 1, 2, 3, 4 vs. surgical category 5 surgical category 3 vs. surgical category 4
Papillary, chromophobe, others	42	1	14	1	9	
Spindle, sarcomatoid	10	0	4	3	2	

Table 3 shows the relationship between levels of tumor thrombus and operative status in 284 patients who were confirmed the level of tumor thrombus in the pretreatment medical records. There were no significant trends between extension of tumor thrombus and operative status, regardless of the metastatic status.

**Table 3:** Correlation between levels of tumor thrombus extension and managed operation

	**Operative status**			**Statistical significance (chi-square test)**
**Level of tumor thrombus**	**Radical nephrectomy and complete resection of thrombus regardless of metastatic status**	**Radical nephrectomy and incomplete resection of thrombus regardless of metastatic status**	**Abandoned operation**	
	**Surgical category 1, 2, 3**	**Surgical category 4**	**Surgical category 5**	
**All patients (n = 284)**				
Intrarenal vein	146	2	4	n.s.
Infrahepatic IVC	95	4	2	
Suprahepatic IVC	20	0	0	
Intrapericardial IVC/ intracardiac extension	9	1	1	
**Patients without apparent distant metastasis (n = 180)**				
Intrarenal vein	99	0	1	n.s.
Infrahepatic IVC	58	2	0	
Suprahepatic IVC	12	0	0	
Intrapericardial IVC/ intracardiac extension	7	1	0	
**Patients without distant metastasis and lymphnode involvement (n = 82)**				
Intrarenal vein	54	0	0	n.s.
Infrahepatic IVC	19	0	1	
Suprahepatic IVC	6	0	0	
Intrapericardial IVC/ intracardiac extension	2	0	0	

**Figure 1:**
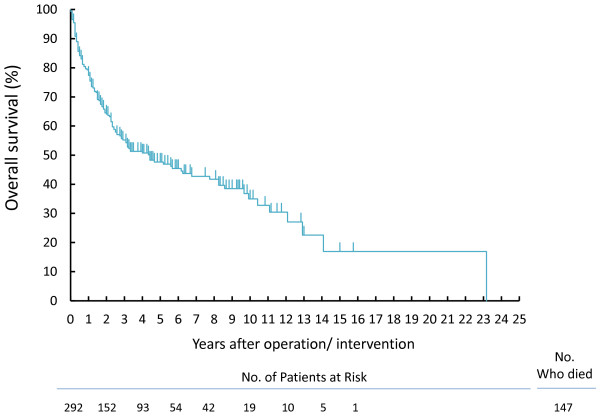
Overall survivals after operation or any interventions in all participants.

The median follow-up was 40.4 months (range; 0 to 278 months). A total of 133 patients died due to RCC and 14 cases due to other causes. Death within one month after operation or diagnosis was seen in 2 (18.2%) of 11 patients who did not undergo operation and in 8 (2.8%) of 281 patients who underwent any operations. Figure 1 shows OS in all participants and the 1-, 3-, 5-year OS and CSS, respectively, were 77.4% and 79.0%, 55.2% and 58.4%, 47.6% and 50.9%. Details of the impacts of pretreatment, treatment, and pathological factors on OS by Kaplan–Meier analyses are shown in Table 4. The operation volume in each hospital, treatment era, and serum calcium concentration were not predictive of OS, but all other pretreatments, treatments, and pathological factors, with the exception of tumor thrombus extension, were significantly associated with OS in univariate analyses. OS and CSS were not significantly different between patients who were treated with and without immune therapies.

**Table 4:** Impacts of various pretreatment, treatment, and pathological factors on overall survival

**Variables**	**All**		**5 years**				**Statistical significance**
	**n**	**Events (all death)**	**No. of patients at risk**	**Events (all death)**	**Cumulative rate(%)**	**S.E.**	
**All cases**	292	147	68	14	47.6%	3.3%	
**Operation volume in each hospital (n)**							
1-9	29	13	3	1	40.9%	14.1%	ns; any comparison
10-19	75	35	21	4	50.4%	6.5%	
20-35	188	99	44	9	46.9%	4.1%	
**Treatment era**							
Before 1999	144	86	56	8	49.2%	4.4%	ns
After 2000	148	61	12	6	43.4%	5.7%	
**Age (years old)**							
30-57	100	49	26	5	48.2%	5.6%	p = 0.00561; Age 58–67 vs. Age 68-87
58-67	96	45	33	5	58.6%	5.5%	
68-87	96	53	9	4	34.2%	6.1%	
**Performance status**							
0	197	82	47	8	55.8%	4.1%	p = 0.00002
1-4	60	41	10	4	25.7%	6.4%	
**Operation**							
Yes	281	136	68	14	49.7%	3.4%	p = 0.00000
No	11	11	0	0			
**ESR (mm/h)**							
Male: 0–49, female: 0-52	91	38	25	5	55.4%	6.0%	p = 0.00024
Male:> = 50, female:> = 56	92	60	12	4	29.4%	5.7%	
**CRP (mg/l)**							
<1.3	129	47	33	5	61.0%	5.0%	p = 0.00003
> = 1.3	126	78	21	7	32.9%	4.9%	
**α2 globulin (%)**							
3.2-10.5	76	27	20	5	60.9%	6.5%	p = 0.00212
10.6-22.3	76	44	15	3	38.4%	6.6%	
**Ca (mg/dl)**							
3.8-9.1	99	49	24	4	49.1%	5.7%	ns
9.2-14.1	100	45	20	4	49.7%	5.9%	
**LDH (U/l)**							
66-288	126	46	21	7	51.1%	6.0%	p = 0.02171
289-1740	125	78	34	6	42.2%	4.7%	
**Hb (g/dl)**							
6.5-11.3	107	64	19	7	32.5%	5.5%	p = 0.00250
11.4-18.5	103	45	27	2	57.7%	5.5%	
**IAP (μg/ml)**							
232-712	102	36	32	5	64.5%	5.3%	p = 0.00000
713-2048	101	65	13	5	29.8%	5.5%	
**T_category**							
= < T3	275	134	68	13	49.8%	3.4%	p = 0.00000
T4	14	12	0	1	9.5%	8.8%	
**N_category**							
N0	118	55	32	6	55.8%	5.0%	p = 0.00000
N1 + N2	61	41	3	4	13.0%	5.9%	
**M_category**							
M0	183	83	44	11	51.3%	4.3%	p = 0.00002
M1	91	61	14	3	31.4%	5.3%	
**Surgical category**							
1	196	82	52	11	55.5%	4.1%	p = 0.00007; surgical category 1 vs. surgical category 3
2	11	6	4	2	52.0%	15.7%	p = 0.00022; surgical category 1 vs. surgical category 4
3	66	41	10	1	34.7%	6.6%	p = 0.00000; surgical category 1 vs. surgical category 5
4	8	7	2	0	25.0%	15.3%	p = 0.00342; surgical category 2 vs. surgical category 5
5	11	11	0	0			p = 0.00112; surgical category 3 vs. surgical category 5
**Tumor size (cm)**							
0-8.3	141	60	41	7	59.6%	4.8%	p = 0.0020
2:8.5-27	139	78	24	7	36.6%	4.7%	
**Pathological subtype**							
Clear cell	206	90	55	11	54.8%	3.9%	p = 0.02816; clear cell vs. papillary, chromophobe, others
Papillary, chromophobe, others	56	31	11	2	41.9%	7.6%	p = 0.00000; clear cell vs. spindle, sarcomatoid
Spindle, sarcomatoid	19	15	1	1	8.3%	7.7%	p = 0.00105; papillary, chromophobe, others vs. spindle, sarcomatoid
**Tumor nuclear grade**							
G1 + G2	206	94	56	12	53.9%	3.9%	p = 0.00215
G3	75	42	12	2	37.1%	6.7%	
**Capsular status**							
Non-invasive	114	55	34	5	52.5%	5.2%	p = 0.00207; non-invasive vs. invasive
Invasive	43	30	6	2	28.4%	7.6%	p = 0.02827; incvasive vs. unknown
Unknown	135	62	28	7	50.2%	5.1%	
**RV/IVC wall invasion**							
Non-invasive	133	52	39	6	59.5%	4.8%	p = 0.00000; non-invasive vs. invasive
Invasive	78	51	15	6	33.5%	6.2%	p = 0.00107; non-invasive vs. unknown
Unknown	81	44	14	2	41.3%	6.4%	
**Tumor thrombus classification 1**							
Renal vein, infrahepatic IVC extension	253	119	63	12	50.9%	3.6%	p = 0.05890
Suprahepatic, intracardial IVC, intracardiac extension	31	22	4	2	28.7%	9.4%	
**Tumor thrombus classification 2**							
Renal vein extension	152	66	41	5	55.1%	4.5%	p = 0.02410; renal vein vs. suprahepatic IVC-intracardiac
Infrahepatic IVC extension	101	53	22	7	44.3%	5.9%	ns; any other comparison
Suprahepatic, intracardial IVC, intracardiac extension	31	22	4	2	28.7%	9.4%	
**Tumor thrombus classification 3**							
Renal vein extension	152	66	41	5	55.1%	4.5%	p = 0.02883
Infrahepatic, suprahepatic, intracardial IVC, intracardiac extension	132	75	26	9	40.6%	5.0%	

surgical category 1; radical nephrectomy and complete resection of thrombus without metastasis, surgical category 2; radical nephrectomy and complete resection of thrombus with metastases that has undergone a cytoreductive surgery, surgical category 3; radical nephrectomy and complete resection of thrombus with unresected metastases, surgical category 4; radical nephrectomy and incomplete resection of thrombus regardless of metastatic status, surgical category 5; abandoned operation.

In terms of the prognostic impact of tumor thrombus extension, a nearly significant and a significant cut-line for predicting OS was between patients with RV or infrahepatic IVC extension and patients with suprahepatic IVC to intracardiac extension (*p* = 0.0589) and between patients with RV extension and patients with infrahepatic IVC to intracardiac extension (*p* = 0.0288). There was no significant difference in OS between patients with RV extension and those with infrahepatic IVC extension.

Using selected factors that significantly predicted OS in Kaplan–Meier analyses, multivariate analyses of independent and significant predictive factors for OS for all patients and for those alive at 1, 2, and 3 years of follow-up were performed using a multivariate Cox proportional hazards model (Table 5). According to multivariate analyses using full-range follow-up data, RV/IVC wall invasion and surgical category were significantly related to OS. According to the partial investigation of survivors at 1 year of follow-up, pathological subtypes and IAP were significantly related to OS. RV/IVC wall invasion was strongly related to OS in restricted survivors at 2 and 3 years of follow-up. The PS was related to OS in restricted survivors at 2 and 3 years of follow-up. Overall, RV/IVC wall invasion was a very significant predictive factor for OS in the full range follow-up and in survivors at 2 and 3 years of follow-up.

**Table 5:** Multivariate Cox proportional hazards model on predictors of overall survival in all participants and yearly-based survivors diagnosed with renal cell carcinoma extending into renal vein or inferior vena cava

**Variables**	**Estimate**	**±**	**Standard error**	**Hazard ratio**		**p value**
				**(95% Confident interval)**		
**All cases**						
Renal vein/ inferior vena cava wall invasion status	0.80	±	0.30	2.22	(1.22-4.02)	0.00876
Pathological subtype	0.45	±	0.24	1.57	(0.97-2.53)	0.06486
Surgical category	0.55	±	0.16	1.73	(1.25-2.39)	0.00088
**Survivors at 1 year of follow-up**						
IAP	2.62	±	0.50	13.68	(5.16-36.3)	0.00000
Pathological subtype	0.53	±	0.21	1.70	(1.11-2.59)	0.01371
**Survivors at 2 years of follow-up**						
Renal vein/ inferior vena cava wall invasion status	1.15	±	0.44	3.16	(1.35-7.44)	0.00825
PS	0.91	±	0.47	2.49	(1.00-6.25)	0.05122
**Survivors at 3 years of follow-up**						
Renal vein/ inferior vena cava wall invasion status	1.60	±	0.48	4.96	(1.93-12.8)	0.00090
PS	0.89	±	0.50	2.43	(0.91-6.44)	0.07531

In the stepwise multiple regression analysis, 232-712 μg/ml IAP, 0 PS, radical nephrectomy and complete resection of thrombus without metastasis in surgical category, non-venous wall-invasive thrombus in renal vein/ inferior vena cava wall invasion, and clear cell subtype on pathological subtype are coded as 1.

Similarly, 713–2048 μg/ml IAP, 1–4 PS, radical nephrectomy and complete resection of thrombus with metastases that has undergone a cytoreductive surgery in surgical category, venous wall-invasive thrombus in renal vein/ inferior vena cava wall invasion, and papillary/chromophobe//others excluding spindle or sarcoma subtype in pathological subtype are coded as 2.

Spindle or sarcomatoid pathological subtype, radical nephrectomy and complete resection of thrombus with unresected metastases in surgical category are coded as 3.

Radical nephrectomy and incomplete resection of thrombus regardless of metastatic status in surgical category is coded as 4.

Abandoned operation in surgical category is coded as 5.

To investigate the impact of follow-up duration on OS, multivariate logistic regression analyses were performed using the restricted data until 1, 2, and 3 years of follow-up (Table 6). Clinicopathologic factors taken into multivariate analyses were selected according to the significance of univariate analyses by Kaplan–Meier methods. Tumor size was a significant predictive factor for OS for a short-term follow-up of within 1 year. RV/IVC wall invasion was significantly correlated with OS if the follow-up duration was restricted to within 1 or 2 years. The surgical category was very strongly correlated with OS in any datasets in which the follow-up duration was restricted to within 1, 2, or 3 years. LDH and α2 globulin were significantly correlated with OS in a restricted follow-up duration of within 2 or 3 years, but were not significant only using restricted datasets within 1 year after interventions.

**Table 6:** Impact of follow-up duration on overall survival in patients with renal cell carcinoma extending into venous system: Multivariate logistic regression analyses using restricted follow-up data until 1, 2, and 3 years after intervention

**Variables**	**Estimate**	**±**	**Standard error**	**Odds ratio**		**p value**
				**(95% Confident interval)**		
**Restricted follow-up until 1 year**						
Surgical category	0.71	±	0.19	2.03	(1.40-2.92)	0.00016
RV/IVC wall invasion status	1.06	±	0.39	2.87	(1.33-6.20)	0.00721
Tumor size	0.69	±	0.40	1.98	(0.91-4.34)	0.08591
Constant	-4.15	±	0.78			
**Restricted follow-up until 2 years**						
LDH	2.48	±	0.94	11.96	(1.91-75.0)	0.00804
Surgical category	1.95	±	0.52	7.04	(2.56-19.4)	0.00016
RV/IVC wall invasion status	1.99	±	0.79	7.28	(1.56-34.0)	0.01152
α2 globulin	1.69	±	0.80	5.44	(1.14-25.9)	0.03351
Constant	-12.10	±	2.86			
**Restricted follow-up until 3 years**						
LDH	1.02	±	0.49	2.78	(1.07-7.25)	0.03621
Surgical category	0.86	±	0.26	2.36	(1.42-3.92)	0.00090
α2 globulin	1.62	±	0.51	5.05	(1.84-13.8)	0.00164
Constant	-8.48	±	1.75			

In the stepwise multiple regression analysis, 3.2-10.5% α2 globulin, 66–288 U/l LDH, radical nephrectomy and complete resection of thrombus without metastasis in surgical category, <8.3-cm tumor size, and non-venous wall-invasive thrombus in RV/IVC wall invasion are coded as 1.

Similarly, 10.6-22.3% α2 globulin, 289–1740 U/l LDH, radical nephrectomy and complete resection of thrombus with metastases that has undergone a cytoreductive surgery in surgical category, 8.5-27.0-cm tumor size, and venous wall-invasive thrombus in RV/IVC wall invasion are coded as 2.

Radical nephrectomy and complete resection of thrombus with unresected metastases, radical nephrectomy and incomplete resection of thrombus regardless of metastatic status, and abandoned operation in surgical category are coded as 3, 4, and 5, respectively.

**Figure 2:**
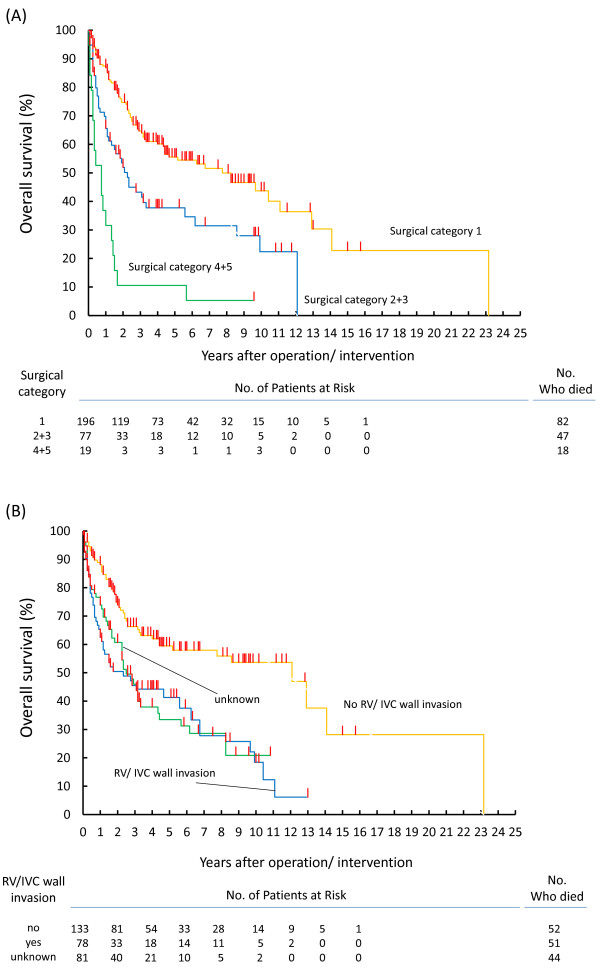
**Overall survivals. (A)** stratified by surgical categories (surgical category 1; radical nephrectomy and complete resection of thrombus without any metastases, surgical category 2 + 3; radical nephrectomy and complete resection of thrombus regardless of cytoreductive surgery at metastatic regions, surgical category 4 + 5; radical nephrectomy and incomplete resection of thrombus regardless of metastatic status or abandoned operation), **(B)** stratified by the status of renal vein (RV) or inferior vena cava (IVC) wall invasion.

The operative and metastatic status/management (i.e.; surgical category) and status of RV/IVC wall invasion were the most universal predictive factors of OS in the present series. Figure 2A shows OS stratified by subdivided surgical categories into complete resection at the origin and thrombus without metastasis (surgical category 1), complete resection at the origin and thrombus with metastases regardless of cytoreductive surgery (surgical category 2 + 3), and incomplete resection at the origin and thrombus or no operation (surgical category 4 + 5). Figure 2B shows OS stratified by presence or absence of RV/IVC wall invasion. Those all subdivided categories shown in Figure 2A and 2B could clearly predict OS.

## Discussion

The prognosis of RCC that has extended into the RV or IVC is comparable to that of RCC without tumor thrombus [10-14]. Skinner *et al*. demonstrated that the 5- and 10-year OS were 55% and 43%, respectively, in 11 patients with non-metastatic RCC with a tumor thrombus that extended into the IVC [12]. Furthermore, Ficarra *et al*. demonstrated that the prognosis of patients with a venous thrombus limited to the subdiaphragmatic IVC was almost identical to that of patients with clinical T2N0M0 disease if there was no perirenal fat invasion, or lymph node or distant metastases [14]. In the present study, 153 patients without apparent or pathological lymph node or distant metastases including tumor thrombi that extended to not only the infrahepatic IVC, but also the suprahepatic IVC, achieved a relatively high 5-year OS of 67.0%. The risk of operation-related death was relatively low at 2.8% in 281 patients who underwent operations.

The relationship between prognosis and the level of tumor thrombus is controversial [1,3,5,9,15-23]. Some reports have demonstrated that there was no relationship between prognosis and level of tumor thrombus [1,3,5,15,17,18,20,21]. In contrast, others indicated differences in prognosis between patients with a tumor thrombus that extended below the diaphragm (or hepatic vein) and those that extended above the diaphragm (hepatic vein) [6,16,22,23], and some investigators have reported that the cut-line for predicting prognosis differed between patients with a tumor thrombus within the RV and those with a thrombus that extended into the IVC [8,9,19]. The controversy regarding the prognostic significance of the level of tumor thrombus may have resulted from differences in the backgrounds of the investigated patients among institutions, progress in the operative technique, mean follow-up duration, and the particular clinicopathologic factors investigated together with the levels of tumor thrombus. In the present study, many available preoperative clinical and pathologic factors were investigated by univariate analyses using the Kaplan–Meier method.

Furthermore, significant factors predicting OS may change according to the follow-up duration, and these differences may result in controversy in terms of the impact of tumor thrombus extension on survival. Therefore, in the present study, multivariate logistic regression analyses of predictors of OS were performed using the restricted follow-up data until 1, 2, and 3 years after intervention against RCC. Overall, fewer global factors were predictive of OS regardless of the follow-up duration, and most independent predictive factors may have changed with follow-up duration. Significant independent predictive factors for a short (within 1 year) and short-to-intermediate (within 1 or 2 years) follow-up period were tumor size and RV/IVC wall invasion, respectively. The surgical category was very strongly correlated with OS in restricted data on follow-up duration within 1, 2, or 3 years. More aggressive tumor removal for origin including venous thrombus might affect survivals. In general, a study investigating the impact of surgery may not include patients with metastatic cases in the cohort. In the present study, a univariate analysis revealed that 5-year all-cause of death in patents with distant metastases was significant higher (68.6%) than that in those without distant metastases (48.7%). However, about a half of patients without distant metastases at the diagnosis was dead mainly due to progressing distant metastases after any intervention. It may means that some patients with venous thrombus may have had invisible distant metastases before surgery. It is also hypothesized that some patients actually did not have any distant metastases at the diagnosis, but unstable tumor cell extending to venous system might disseminate during surgical intervention or residual tumor cells in the venous wall or perirenal fat following surgery might be an origin of progressing distant metastases during long-term follow-up. Therefore, we did not exclude patients with visible distant metastases in the present study. Then, a multivariate Cox proportional hazards model showed that presence or absence of visible distant metastases at the diagnosis was not an independent prognostic factor to predict OS in patients with renal cell carcinoma extending into venous system. The level of tumor thrombus setting the cut-line between patients with a thrombus that extended within the RV and those with a thrombus that extended into the IVC was included in the multivariate logistic regression analyses, but it was not an independent predictive factor for OS using any restricted follow-up duration.

For postoperative follow-up at an outpatient clinic, it would be useful to investigate changes in predictive factors with elapsed time after intervention. In the present study, multivariate Cox proportional hazards models were used to investigate predictors of OS not only using the full-range follow-up data of all participants, but also those of survivors at 1, 2, and 3 years of follow-up. RV/IVC wall invasion was significantly related to OS in full-range follow-up data and in survivors at 2 and 3 years of follow-up. The surgical category was significantly related to OS using unrestricted datasets. Investigation of survivors at 1, 2, and 3 years of follow-up showed that many independent predictive factors were not universal at any follow-up period and changed accordingly. For example, IAP was very strongly related to OS in survivors at 1 year of follow-up, but not predictive in limited survivors at 2 and 3 years of follow-up.

According to the Union for International Cancer Control (UICC) TNM classification published in 2002, RCC with a tumor thrombus was classified as T3b or T3c regardless of the presence or absence of perirenal fat invasion or adrenal direct invasion. Recently, some investigators demonstrated the independent and positive impact of adrenal, perirenal fat, or renal sinus invasion on survival [23-28]. In the present series, only 157 (53.8% of all participants) patients had data on the status of renal capsular invasion. Although the present study could not demonstrate independent significance of the presence of renal capsular invasion on OS, the significance of local invasion on survival in RCC extending into venous systems cannot be concluded.

A wide-range registration period during 30 years may be a flaw of the present study because diagnostic quality of CT scan especially in 8 patients diagnosed in 1980’s could not be enough. Those 8 patients diagnosed in 1980’s were followed until between 1994 and 2003 (during 145 months in median). Therefore, recurrence after intervention could be checked using more reliable CT imaging with time. Among 8 patients, 7 were diagnosed after 1987 and the oldest patient was diagnosed in 1980. The oldest case was dead due to progressing distant metastases, but was alive until the end of 2003. Therefore, we did not exclude those important patients diagnosed in 1980’s and followed for a long-term from the present study. The present retrospective multi institutional study included some flaws in the quality of database. There were some missing data on pretreatment clinical factors and pathological findings. However, there were only 6 factors (e.g.; ESR, α2 globulin, Ca, IAP, N category in TNM classification and capsular status) that included 30% or more unknown data in each among 22 pretreatment, treatment and pathological factors using Kaplan-Meier analysis on overall survival (shown in Table 4). Lacking restricted follow-up strategy could be a flaw of the present retrospective study. If a surrogate endpoint such as progression-free survival was set as a primary endpoint, likelihood of misclassification of the event would not be ignored. However, overall survival was set as a primary endpoint in the present study. Therefore, likelihood of misclassification of the event may not affect the conclusions and could not be a serious flaw in the present study.

In the present series, all follow-up data were retrospectively registered in the era before introduction of molecular-targeted therapy in Japan. It is necessary to investigate the usefulness of neoadjuvant, adjuvant, or salvage molecular targeted therapy in a nationwide or international prospective study. Furthermore, the present finding on the importance of RV/IVC invasion certainly raises important questions on the need of IVC excision in patients with tumor thrombus invading to venous wall and for imaging techniques to be improved so that IVC invasion can be identified pre-operatively. The predictive impact of modern imaging technique such as multi-slice CT and 3-tesla magnetic resonance imaging (MRI) on likelihood of venous wall invasion and prognostic impact of following IVC excision should be also prospectively investigated in the future.

## Conclusions

In patients diagnosed with an advanced stage of RCC with a tumor thrombus, many important independent predictive factors were identified in each subgroup analysis in terms of restricted follow-up duration and yearly-based survivors. However, the most universal factors affecting improvement of both in short-term and long-term survivals in RCC with venous thrombus could be feasible aggressive cytoreductive operation and absence of venous wall invasion even in a case of metastatic disease. It may mean that the best available cytoreductive operation following more reliable preoperative imaging for predicting venous wall invasion status would improve OS for patients with advanced RCC extending into venous systems.

## Competing interests

Any authors have no competing interest that could be perceived as prejudicing the impartiality of the research reported and does not have any financial supports from industrial companies that are related with this research.

## Authors’ contributions

MS, MK and KI participated in the design of the study and performed the statistical analysis. TTsushima, WO, NS, KI, ME, TTakayama, YF, MN, GK, TK, MT, NM, YN, TA participated in the acquisition of clinicopathologic data and carried out outcome research. TF and SN participated in revising the draft critically for important intellectual content. All authors read and approved the final manuscript.

## Pre-publication history

The pre-publication history for this paper can be accessed here:

http://www.biomedcentral.com/1471-2407/13/447/prepub
